# Grants to Hospitals Recommended by the Distribution Committee

**Published:** 1918-12-21

**Authors:** 


### KING EDWARD'S HOSPITAL FUND—(*continued*).

### Grants to Hospitals Recommended by the Distribution Committee.

*The Distribution Committee desire to draw attention to the fact that it must not be assumed that the reduction or absence of a grant implies dissatisfaction.*

Acton Hospital, £200. Alexandra Hospital for Children, £200. Beckenham Cottage Hospital, £50, of which £25 to improvements. Belgrave Hospital for Children, £1,000. Blackheath and Charlton Hospital, £150. Bolingbroke Hospital, £1,000, of which £250 to reduce debt. British Hospital for Mothers and Babies, Woolwich, £250, of which £50 to recent improvements. A grant of £1,500 was set aside in 1913 towards the building of the amalgamated hospital, in accordance with scheme to be approved. Canning Town Women's Settlement Hospital (late Medical Mission), £550, of which £50 to reduce debt. Central London Ophthalmic Hospital, £500. Central London Throat and Ear Hospital, £200. (See paragraph 8 of Report). Charing Cross Hospital, £3,500. Chelsea Hospital for Women, £2,000, of which £1,500 to reduce debt. Cheyne Hospital for Sick and Incurable Children, £300. City of London Hospital for Diseases of the Chest (Victoria Park), £5,500, of which £2,000 to reduce deficit on recent improvements in accordance with the scheme submitted to the Fund. City of London Maternity Hospital, £1,000, of which £250 to reduce general fund debt. Clapham Maternity Hospital, £1,250, of which £450 to reduce debt. Dreadnought Hospital, £3,500. East End Mothers' Lying-in Home, £750. East Ham Hospital, £100. East London Hospital for Children, £3,500. Elizabeth Garrett Anderson Hospital (formerly New Hospital for Women), £1,000. Eltham and Mottingham Cottage Hospital, £100. Finchley Cottage Hospital, £125. Florence Nightingale Hospital for Gentlewomen, £300, of which £100 to reduce debt. French Hospital, £200. General Lying-in Hospital, £1,350, of which £1,000 to deferred scheme of improvements. Great Northern Central Hospital, £6,000, of which £1,000 to reduce debt, and £1,000 to deferred scheme for new nurses' home. Grosvenor Hospital for Women, £500. Guy's Hospital, £11,000, of which £1,000 to reduce deficit on recent building improvements, and £1,000 to deferred improvements in nursing accommodation, in accordance with the schemes submitted to the Fund. Hampstead General and North-West London Hospital, £3,550, of which £150 as agreed upon amalgamation with North-West London Hospital, and £1,500 in reduction of debt. Hornsey Cottage Hospital, £50. Hospital and Home for Incurable Children, Hampstead, £50, in consideration of the fact that curable cases are admitted. Hospital and Home for Sick Children, Sydenham, £400. Hospital for Consumption, Brompton (including Sanatorium at Frimley), £5,500. Hospital for Diseases of the Throat, £1,400, of which £900 is maintenance grant, payable, as agreed, under the terms of the amalgamation with the London Throat Hospital, and £500 is a grant towards loss incurred by postponement of building scheme through the war. Hospital for Epilepsy and Paralysis, £1,750, of which £250 to reduce debt. Hospital for Sick Children, £5,500, of which £1,500 to reduce debt. Hospital for Women (Soho Square), £2,500, of which £1,000 to reduce debt. Hospital of St. John and St. Elizabeth, £500. Infants Hospital, £750, of which £250 to reduce debt. Italian Hospital, £400. Kensington and Fulham General Hospital, £100, of which £25 to recent improvements. Kensington Dispensary and Children's Hospital, £50. King Edward Memorial Hospital (Ealing), £750. King's College Hospital, £8,000, of which £5,000 to reduction of debt on removal to South London, making £72,100 in all contributed by the Fund to this purpose. Leyton, Walthamstow, and Wanstead Children's and General Hospital, £650. London Fever Hospital, £200. London Homœopathic Hospital, £1,750, of which £500 to reduce general fund debt. London Hospital, £14,500, of which £1,500 to new nurses' home in accordance with the scheme submitted to the Fund. London Lock Hospital, £2,500, of which £1,000 to maintenance of Harrow Road Hospital, and £1,500 to deferred scheme of reconstruction. London Temperance Hospital, £1,750. Maternity Charity and

**KING EDWARD'S HOSPITAL FUND—*(continued)*.**

District Nurses' Home, Plaistow, £400. Memorial Hospital, Mildmay Park, £450, of which £200 to reduce debt. Metropolitan Hospital, £4,000, of which £1,000 to reduce debt. Middlesex Hospital, £7,500, of which £1,000 to reduce debt, and £1,000 to recent improvements. Middlesex Hospital Cancer Charity, £200. Mildmay Mission Hospital, £550. Miller General Hospital for South-East London, £1,800. Mothers' Hospital of the Salvation Army, £450. National Hospital for Diseases of the Heart, £300. National Hospital for the Paralysed and Epileptic, £4,000, of which £500 to reduce bank loan. Nelson Hospital (late South Wimbledon, etc.), £500. Norwood Cottage Hospital, £350. Paddington Green Children's Hospital, £1,000, of which £150 to improvements. Passmore Edwards Hospital for Willesden, £150. Passmore Edwards Hospital for Wood Green, etc., £100, of which £50 to reduce debt. Poplar Hospital for Accidents, £200. Prince of Wales's General Hospital, £5,000, of which £2,000 to reduce general fund debt, and £500 to improvements to lift. Queen Charlotte's Lying-in Hospital, £2,250, of which £250 to reduce debt, and £750 to deferred scheme of improvements. Queen Mary's Hospital for the East End, incorporating West Ham and Eastern General Hospital, £5,000, of which £1,000 to scheme of improvements to be submitted to the Fund. Queen's Hospital for Children, £4,750, of which £500 to reduce debt, and £1,250 to deferred scheme for new out-patient department. Royal Dental Hospital for London, £450. Royal Eye Hospital, £600. Royal Free Hospital, £2,500. Royal Hospital for Diseases of the Chest (City Road), £200. Royal Hospital, Richmond, £400. Royal London Ophthalmic Hospital, £2,500. Royal National Orthopædic Hospital, £750. Royal Waterloo Hospital for Children and Women, £3,000, of which £1,000 to reduce secured debt. Royal Westminster Ophthalmic Hospital, £150. St. Andrew's Hospital (Dollis Hill), £50. St Columba's Hospital, £350. St. George's Hospital, £4,500. St. John's Hospital, Lewisham, £600. St. Luke's Hospital for Advanced Cases, £200. St. Mark's Hospital, £350. St. Mary's Hospital, £3,500. St. Mary's Hospital for Women and Children, Plaistow, £2,600, of which £500 to recent temporary improvement in nurses' and servants' quarters and out-patient department in old hospital building, and £1,000 to deferred scheme of rebuilding. St. Monica's Home Hospital, £300, of which £150 to reduce debt. St. Peter's Hospital for Stone, £400, of which £100 to reduce deficit on fire escape staircases. St. Saviour's Hospital for Ladies of Limited Means (Osnaburgh Street), £200. St. Thomas's Hospital, £1,000. Samaritan Free Hospital for Women, £1,600, of which £350 towards improved access for nurses to wards. Santa Claus Home, £50. Stoke Newington Home Hospital for Women, £100. University College Hospital, £7,000, of which £500 to improvements to lifts, and £500 to the scheme of amalgamation with the Royal Ear Hospital when approved, in addition to £500 out of the amalgamation grants set aside in former years. (See paragraph 8 of Report.) Victoria Hospital for Children, £1,500. West End Hospital for Nervous Diseases, £500, of which £100 to improvements in electrical apparatus. Western Ophthalmic Hospital, £600, of which £500 to reduction of mortgage debt. West London Hospital. £5,500, of which £1,500 to improvements in accordance with scheme to be submitted to the Fund. Westminster Hospital, £5,800, of which £2,000 to reduce general fund debt, and £300 annual grant to acquisition of new site as agreed. Wimbledon Hospital, £100. Woolwich and Plumstead Cottage Hospital, £25. (See paragraph 7 of Report.) Special grant: Woolwich and District War Memorial Hospital Building Fund, £5,000. (See paragraph 7 of Report.)

**SUMMARY.**

| | |
|---|---|
| **Grants** ... ... ... ... ... ... | **£185,000** |
| **Special Grant** ... ... ... ... ... | **5,000** |
| **Total Grants to Hospitals for the year 1918** ... ... ... ... ... ... | **£190,000** |

